# Competitive Inhibition of Okanin against *Plasmodium falciparum* Tyrosyl-tRNA Synthetase

**DOI:** 10.3390/ijms25094751

**Published:** 2024-04-26

**Authors:** Guangpu Yang, Yali Liang, Xiang Li, Zan Li, Yinying Qin, Qilu Weng, Yujuan Yan, Yijun Cheng, Yunan Qian, Litao Sun

**Affiliations:** 1 School of Public Health (Shenzhen), Shenzhen Campus of Sun Yat-sen University, Sun Yat-sen University, Shenzhen 518107, China; yangguangpu183@163.com (G.Y.); liangyali2023@163.com (Y.L.); jnmclixiang@126.com (X.L.); lizan6@mail2.sysu.edu.cn (Z.L.); qinyy8@mail2.sysu.edu.cn (Y.Q.); wengqlu@mail2.sysu.edu.cn (Q.W.); yanyj29@mail2.sysu.edu.cn (Y.Y.); chengyj28@mail2.sysu.edu.cn (Y.C.); 2 Key Laboratory of Molecular Virology and Immunology, Shanghai Institute of Immunity and Infection, Chinese Academy of Sciences, Shanghai 200031, China; 3 Shenzhen Key Laboratory of Pathogenic Microbes and Biosafety, Shenzhen 518107, China

**Keywords:** malaria, tyrosyl-tRNA synthetase, okanin, drug screening

## Abstract

Malaria is a severe disease that presents a significant threat to human health. As resistance to current drugs continues to increase, there is an urgent need for new antimalarial medications. Aminoacyl-tRNA synthetases (aaRSs) represent promising targets for drug development. In this study, we identified *Plasmodium falciparum* tyrosyl-tRNA synthetase (*Pf*TyrRS) as a potential target for antimalarial drug development through a comparative analysis of the amino acid sequences and three-dimensional structures of human and plasmodium TyrRS, with particular emphasis on differences in key amino acids at the aminoacylation site. A total of 2141 bioactive compounds were screened using a high-throughput thermal shift assay (TSA). Okanin, known as an inhibitor of LPS-induced TLR4 expression, exhibited potent inhibitory activity against *Pf*TyrRS, while showing limited inhibition of human TyrRS. Furthermore, bio-layer interferometry (BLI) confirmed the high affinity of okanin for *Pf*TyrRS. Molecular dynamics (MD) simulations highlighted the stable conformation of okanin within *Pf*TyrRS and its sustained binding to the enzyme. A molecular docking analysis revealed that okanin binds to both the tyrosine and partial ATP binding sites of the enzyme, preventing substrate binding. In addition, the compound inhibited the production of *Plasmodium falciparum* in the blood stage and had little cytotoxicity. Thus, okanin is a promising lead compound for the treatment of malaria caused by *P. falciparum*.

## 1. Introduction

Malaria is a major threat to public health, particularly in tropical regions and countries. This life-threatening disease is caused by parasitic infections from single-celled organisms of the genus Plasmodium, including *Plasmodium falciparum* (*P. falciparum*), *Plasmodium knowlesi* (*P. knowlesi*), *Plasmodium vivax* (*P. vivax*), *Plasmodium malariae* (*P. malariae*), and *Plasmodium ovale* (*P. ovale*) [1,2]. Among these, *P. falciparum* is associated with the most severe cases. In 2022, an estimated 249 million malaria cases resulted in 608,000 deaths worldwide, mostly in sub-Saharan Africa [3]. Progress has been made in reducing global malaria morbidity and mortality with the support of the World Health Organization (WHO) [3]. Interventions such as long-lasting insecticide-treated nets, early detection, and effective treatments such as artemisinin-based combination therapies (ACTs) are being implemented. However, half of the world’s population remains at risk, with approximately 3.1 billion people living in malaria-endemic areas (tropical and subtropical) [4]. Notably, drug-resistant *P. falciparum* parasites are emerging in many regions and countries [5]. Increasing resistance to artemisinin-based combination therapies, the first-line treatment for *P. falciparum*, threatens the long-term viability of recent advances in malaria control and the possibility of eradicating malaria. Therefore, new chemical scaffold drugs are a top priority for antimalarial development.

Screening for drugs that target essential parasite proteins, such as aminoacyl-tRNA synthetases (aaRSs), is a valuable strategy in drug discovery [6]. AaRSs are vital enzymes in all cellular life, essential for the translation of the genetic code [7,8]. *P. falciparum* has 37 aaRS genes, encoding 36 enzymes, with 16 in the cytoplasm, 15 in the apicoplast, and 4 in both locations [9]. They are promising targets for the development of antibacterial, antifungal, and antiparasitic drugs, as well as for the treatment of other human diseases [7,10,11]. For instance, borrelidin, an inhibitor of threonyl-tRNA synthetase (ThrRS), exhibited potent antimalarial activity against drug-sensitive and drug-resistant parasite strains while exhibiting minimal cytotoxicity against human cells [12,13]. Another example is febrifugine, a specific prolyl-tRNA synthetase (ProRS) inhibitor, which displayed high efficacy against both liver and asexual blood stages of the malaria parasite and was validated in the *P. berghei* mouse model of malaria [14]. These examples demonstrate the efficacy of targeting tyrosyl-tRNA synthetase (TyrRS) for the development of antimalarial drugs.

Here, we performed a systematic analysis of amino acid sequence alignment and three-dimensional structural comparison of the human and plasmodium aaRS families. Remarkably, compared to Homo sapiens tyrosyl-tRNA synthetase (*Hs*TyrRS), which has evolved a novel EMAP II-like domain with functionality in mononuclear phagocytes and polymorphonuclear leukocytes, *P. falciparum* TyrRS (*Pf*TyrRS) consists only of the catalytic and anticodon-binding domains. The sequence divergence between the catalytic domains of *Hs*TyrRS and *Pf*TyrRS is evident, with X-ray crystallography revealing differential flexibility in a loop at the catalytic site between the two [15]. As a result, *Pf*TyrRS emerges as a promising target for antimalarial drug development.

Using *Pf*TyrRS as a target, we employed the thermal shift assay (TSA), a high-throughput compatible assay, to screen 2141 natural bioactive compounds for interactions. Through ATP hydrolysis experiments, we identified okanin as a natural compound that inhibits *Pf*TyrRS. Subsequent studies using bio-layer interferometry (BLI) and molecular dynamics (MD) simulations evaluated the affinity and conformational stability between okanin and *Pf*TyrRS. Furthermore, molecular docking methods elucidated the molecular mechanism underlying the inhibition of *Pf*TyrRS by okanin. Finally, the antimalarial activity of okanin was verified in vitro. In conclusion, our study reports the discovery of a novel aaRS inhibitor, okanin, which provides a promising chemical scaffold for antimalarial drug development and highlights the potential of repurposing kinase-inhibiting drugs as aaRS inhibitors for the treatment of human diseases.

## 2. Results

### 2.1. PfTyrRS Is a Promising Target for Antimalarial Drug Development

**Figure 1:**
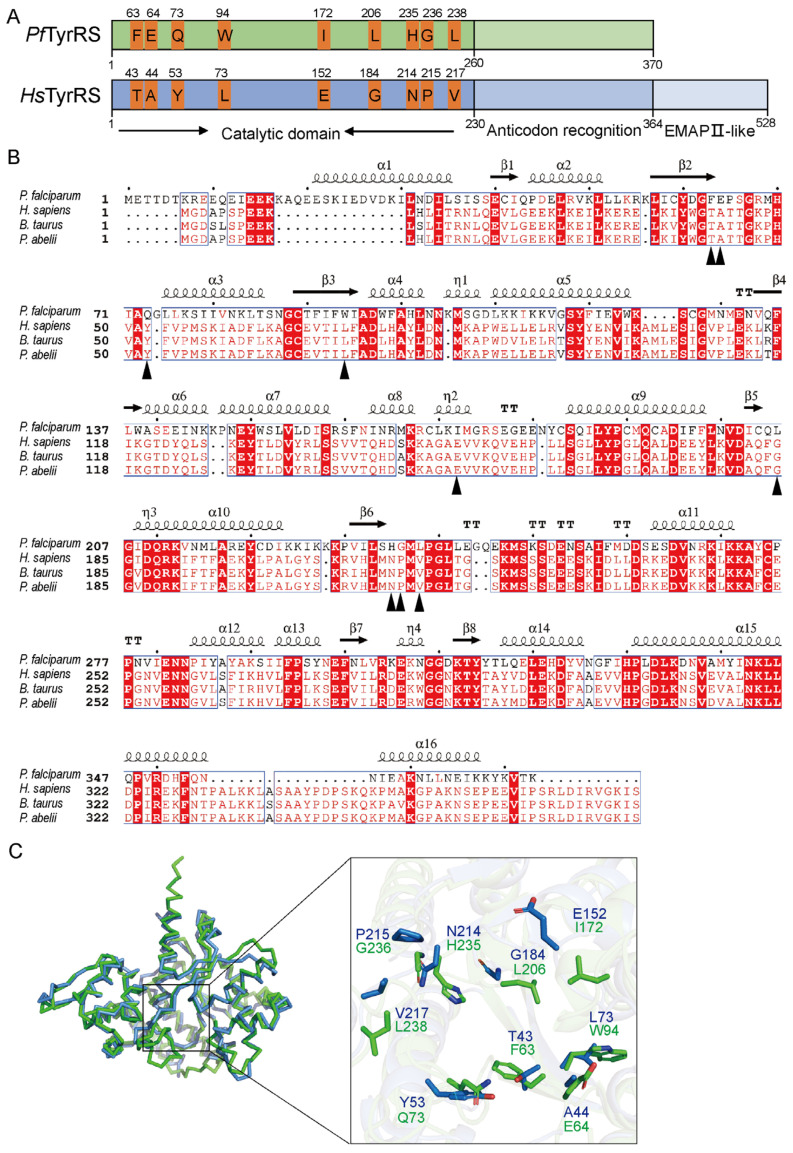
Sequence and structural analysis of *Pf*TyrRS. (**A**) *Pf*TyrRS and *Hs*TyrRS sequence alignment model. (**B**) Sequences are shown for *Plasmodium falciparum* (*P. falciparum*), *Homo sapiens* (*H. sapiens*), *Bos taurus* (*B. taurus*), and *Pongo abelii* (*P. abelii*). Alignments were generated using ESPript (https://espript.ibcp.fr/, accessed on 20 March 2024). The red background color indicates that the amino acid at this site is relatively conserved. The triangles indicate key amino acid sites where *Hs*TyrRS differs from *Pf*TyrRS. Identical and similar amino acid residues are shown in red and white, respectively. Bule frames represent residues with similarity at that position. The black dots represent every ten amino acid positions in *Pf*TyrRS. (**C**) Structural analysis between *Pf*TyrRS and *Hs*TyrRS. The *Pf*TyrRS is presented as a cartoon in green; *Hs*TyrRS is presented in sky blue; Oxygen atoms are shown in red.

To determine the differences between human and *plasmodium* TyrRS, we performed an amino acid sequence analysis of *Pf*TyrRS. In contrast to *Hs*TyrRS (Sequence ID: NP_003671.1), which has evolved a novel EMAP II-like domain with functionality in mononuclear phagocytes and polymorphonuclear leukocytes, *Pf*TyrRS (Sequence ID: XP_001349444.1) consists only of the catalytic and anticodon-binding domains (Figure 1A). Further, we performed a sequence alignment of *Pf*TyrRS with several higher eukaryotic organisms, such as *Homo sapiens* (*H. sapiens*), *Bos taurus* (*B. taurus*) (Sequence ID: NP_776645.1), and *Pongo abelii* (*P. abelii*) (Sequence ID: NP_001126056.1), which revealed a relatively low sequence conservation (Figure 1B). There is only about 30% homology between *Pf*TyrRS and other organisms in the catalytic and anticodon-binding domains (Figure 1B). In addition, a three-dimensional structural comparison of the catalytic pockets of *Pf*TyrRS (PDB ID, 3VGJ) and *Hs*TyrRS (PDB ID, 4QBT) was performed (Figure 1C) [16,17]. This analysis identified 24 amino acid sites in the *Pf*TyrRS catalytic pocket that interact with 5′-O-[N-(L-tyrosine) sulfonamyl] adenosine (Tyr-AMS), with 9 amino acids differing from those of *Hs*TyrRS (Figure 1C). Taken together, these observations highlight *Pf*TyrRS as a promising target for antimalarial drug development.

**Figure 2:**
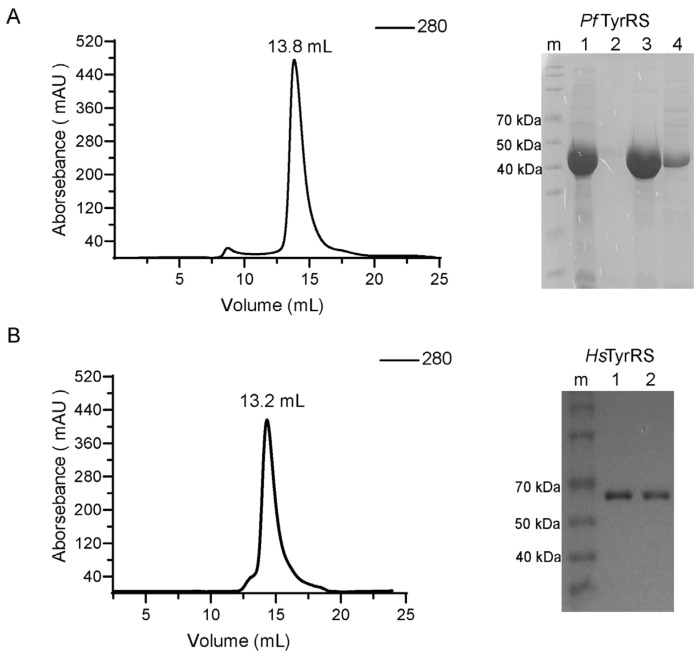
Purification and identification of recombinant *Pf*TyrRS and *Hs*TyrRS. (**A**) Gel filtration, using a Superdex 200 Increase 10/300 GL column, was used as a final purification step for *Pf*TyrRS. SDS-PAGE analysis of purified recombinant *Pf*TyrRS was performed using a 12% acrylamide gel. m: protein maker, 1: loading sample, 2: pre-peak, 3: mid-peak, 4: post-peak. (**B**) Gel filtration, using a Superdex 200 Increase 10/300 GL column, was used as a final purification step for *Hs*TyrRS. SDS-PAGE analysis of purified recombinant *Hs*TyrRS was performed using a 12% acrylamide gel. m: protein maker, 1–2: purified protein of *Hs*TyrRS.

The recombinant constructs of *Pf*TyrRS and *Hs*TyrRS were expressed in *Escherichia coli* (*E. coli*) and subsequently purified using Ni-column chromatography, an ion exchange Q HP column, and size exclusion chromatography. An SDS-PAGE analysis showed that the *Pf*TyrRS protein has an approximate molecular weight of 47 kDa. Additionally, it was observed that when eluted as a single peak at 13.8 mL volume, it corresponded to an apparent molecular weight of a homodimer, approximately 94 kDa (Figure 2A). Similarly, the SDS-PAGE analysis revealed that the *Hs*TyrRS protein has an approximate molecular weight of 64 kDa. Upon elution, the protein was observed as a single peak at 13.2 mL volume, corresponding to an apparent molecular weight of a homodimer, approximately 128 kDa (Figure 2B). Following purification, the proteins were concentrated for further experimentation.

### 2.2. Multi-Approach Drug Screening Based on PfTyrRS

**Figure 3:**
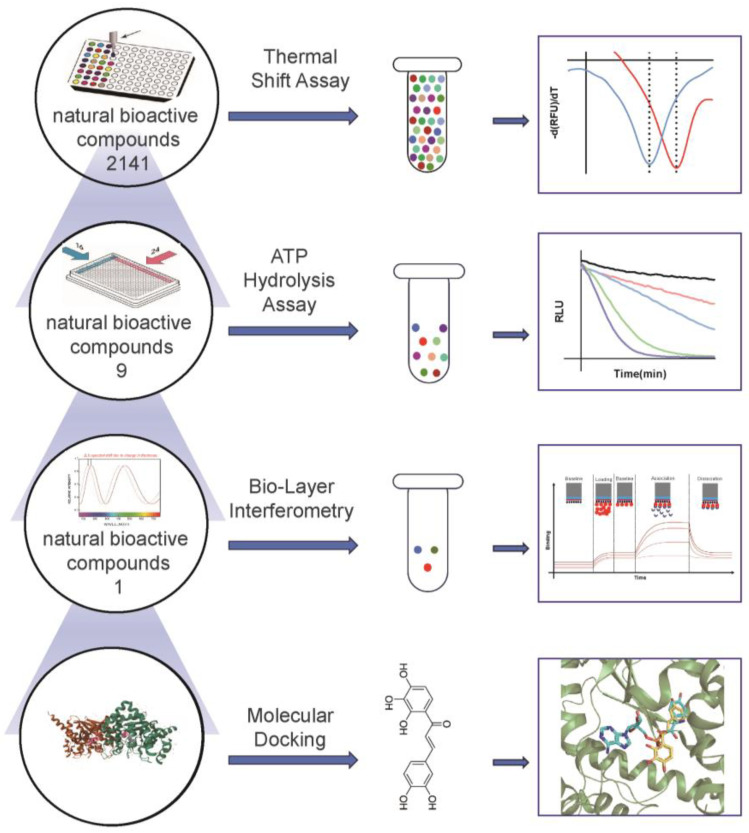
Flow chart of antimalarial drug screening.

To identify the potential molecules targeting *Pf*TyrRS, we utilized a series of assays including the TSA, ATP hydrolysis assay, BLI, and molecular docking to screen for candidate compounds. The TSA enabled the identification of compounds that bind to *Pf*TyrRS, while the ATP hydrolysis assay was used to assess their impact on *Pf*TyrRS enzymatic activity. BLI provided insights into the binding kinetics and affinity of candidate compounds for *Pf*TyrRS, while molecular docking simulations elucidated their binding modes and inhibitory mechanisms. Through these experiments, we identified one or several candidate compounds that exhibited promising inhibitory effects on *Pf*TyrRS stability, enzymatic activity, and binding affinity. These findings lay the foundation for the development of novel antimalarial therapies targeting *Pf*TyrRS and highlight the potential of aminoacyl-tRNA synthetase inhibitors for the treatment of malaria. A workflow chart is shown in Figure 3.

### 2.3. TSA-Based Screening for Potential PfTyrRS Inhibitors

**Figure 4:**
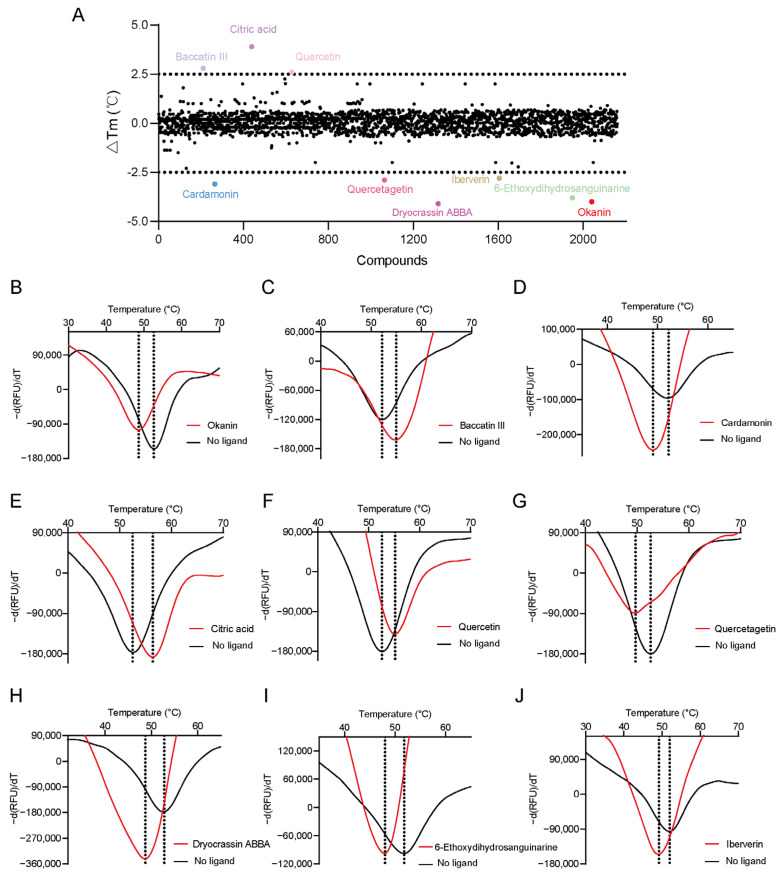
High-throughput screening for *Pf*TyrRS inhibitors based on the thermal shift assay. (**A**) Screening for *Pf*TyrRS inhibitors based on the thermal shift assay. A total of 9 compounds shift the melting temperature of *Pf*TyrRS by >2.5 °C and are considered positive hits among 2141 tested compounds. Black points represent negative hits. (**B**–**J**) The interaction analysis of *Pf*TyrRS and 9 positive compounds by TSA. The Tm of *Pf*TyrRS was approximately 52.5 °C and a shift > 2.5 °C was observed in the Tm with okanin, baccatin III, cardamonin, citric acid, quercetin, quercetagetin, dryocrassin ABBA, 6-ethoxydihydrosanguinarine, and iberverin.

The recombinant *Pf*TyrRS protein was used as the bait in a fluorescence-based TSA. In this assay, as the temperature increases, the protein undergoes thermal unfolding, exposing its hydrophobic core. Consequently, a fluorescent dye binds to these hydrophobic regions, resulting in fluorescence. The fluorescence intensity is closely monitored, and the temperature at which the protein reaches its midpoint unfolding transition is defined as the Tm. Ligand binding often alters protein thermal stability during denaturation, allowing the assessment of potential small-molecule binding by examining the shift in the Tm [18,19]. Within this context, 2141 natural bioactive compounds were screened (Figure 4A, Supplementary Materials, Table S1). Dimethyl sulfoxide (DMSO) was used as a negative control. The Tm of the *Pf*TyrRS in the absence of any compound was 52.5 °C. The assay identified compounds that shifted the Tm of *Pf*TyrRS by >2.5 °C as positive hits (Figure 4B–J). Finally, the TSA assay identified nine positive hits, including okanin, baccatin III, cardamonin, citric acid, quercetin, quercetagetin, dryocrassin ABBA, 6-ethoxydihydrosanguinarine, and iberverin (Table 1). These compounds were further subjected to enzymatic assays.

### 2.4. Okanin Binds and Inhibits ATP Hydrolysis of PfTyrRS

**Figure 5:**
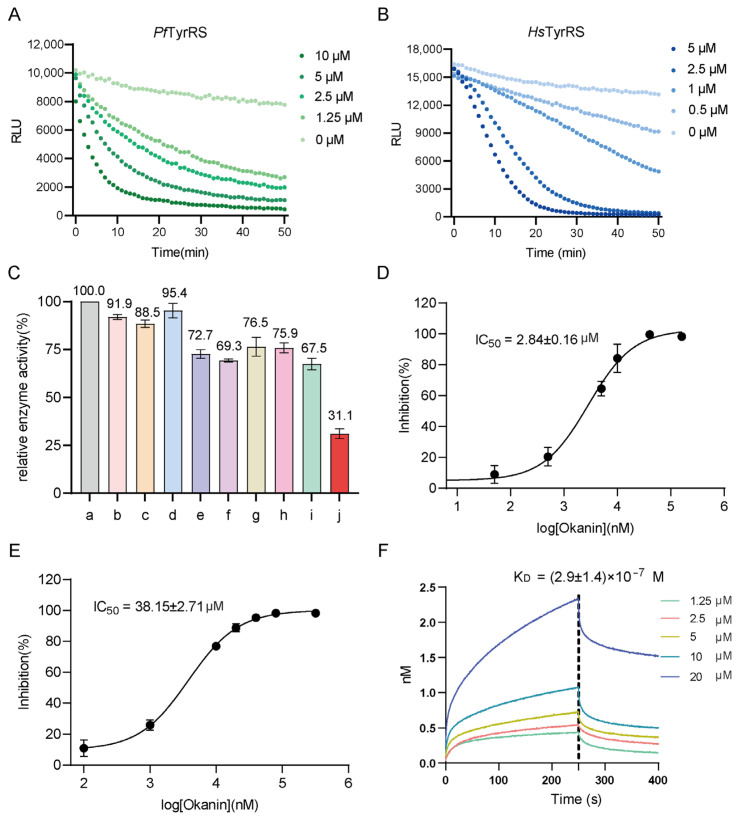
Okanin is a novel *Pf*TysRS inhibitor. (**A**) ATP hydrolysis assay of *Pf*TyrRS. The luminescence of the reaction mixture with time was measured by standard ATP at different *Pf*TyrRS concentrations. (**B**) ATP hydrolysis assay of *Hs*TyrRS. The luminescence of the reaction mixture with time was measured by standard ATP at different *Hs*TyrRS concentrations. (**C**) Inhibition activity of 9 different compounds based on the ATP hydrolysis assay. Okanin shows a good inhibitory effect, while the other 8 compounds show no significant inhibitory effect. Error bars indicate standard deviations. a: no ligand, b: baccatin III, c: cardamonin, d: citric acid, e: quercetin, f: quercetagetin, g: dryocrassin ABBA, h: 6-ethoxydihydrosanguinarine, i: iberverin, j: okanin. (**D**) Okanin shows efficient inhibitory activity of *Pf*TyrRS in vitro. *Pf*TyrRS was incubated with different concentrations of okanin. IC_50_ was calculated based on the inhibition assay. Data represent the average of three independent assays and error bars correspond to SD. (**E**) Okanin shows weak activity against *Hs*TyrRS in vitro. *Hs*TyrRS was incubated with different concentrations of okanin. IC_50_ was calculated based on the inhibition assay. Data represent the average of three independent assays, and error bars correspond to SD. (**F**) Binding sensorgrams for the interaction of okanin with immobilized *Pf*TyrRS. The five curves are generated from 20, 10, 5, 2.5, and 1.25 μM okanin from top to bottom.

We employed an ATP detection kit to assess the ATP consumption capacity of recombinant *Pf*TyrRS and *Hs*TyrRS during the initial phase of the aminoacylation process. The kit functions on the basis of firefly luciferase catalyzing the conversion of luciferin into fluorescence, utilizing ATP as the energy substrate. The resultant fluorescence production is directly proportional to the ATP concentration present, thereby allowing for accurate quantification. In Figure 5A, we present the temporal variation in luminescence levels in a standard ATP assay reaction mixture in the presence of varying concentrations of *Pf*TyrRS (0 µM, 1.25 µM, 2.5 µM, 5 µM, and 10 µM). Figure 5B illustrates a similar depiction for *Hs*TyrRS concentrations (0 µM, 0.5 µM, 1 µM, 2.5 µM, and 5 µM). Therefore, we selected 1.25 µM *Pf*TyrRS and 1 µM *Hs*TyrRS for the subsequent enzyme activity inhibition experiment. We evaluated nine natural bioactive compounds for their ability to inhibit *Pf*TyrRS ATP hydrolysis. Each compound was incubated with *Pf*TyrRS for 30 min at room temperature, followed by ATP hydrolysis experiments. Nine compounds do not directly inhibit the ATP luciferase assay. Okanin exhibited the most significant inhibitory effect, with a 68.9% inhibition rate (Figure 5C). To quantify the inhibitory activity of okanin, we performed an enzyme kinetics analysis, which revealed an IC_50_ of 2.84 ± 0.16 μM for *Pf*TyrRS (Figure 5D). Additionally, we evaluated the inhibitory effect of okanin on *Hs*TyrRS ATP hydrolysis activity and observed an IC_50_ of 38.15 ± 2.71 μM (Figure 5E). The difference in the inhibition rate of okanin on *Pf*TyrRS and *Hs*TyrRS suggests that okanin or its derivatives can be used as a potential therapeutic and preventive agent for *P. falciparum*.

**Figure 6:**
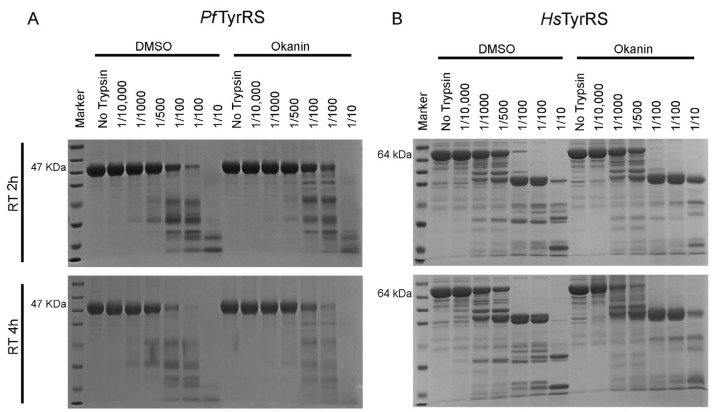
Limited proteolysis analysis. (**A**) The *Pf*TyrRS proteins (in the presence and absence of okanin) were incubated with trypsin at different concentrations for 2 h and 4 h before the reactions were quenched, and the products were separated by SDS-PAGE. (**B**) The *Hs*TyrRS proteins (in the presence and absence of okanin) were incubated with trypsin at different concentrations for 2 h and 4 h before the reactions were quenched, and the products were separated by SDS-PAGE.

To further validate the binding kinetics and affinity between okanin and *Pf*TyrRS, we performed BLI assays. BLI is an optical technique that analyzes the interference pattern of white light reflected from two surfaces: a layer of immobilized protein on a biosensor tip and an internal reference layer, allowing real-time monitoring of changes in molecular binding. The BLI study showed that okanin has a high affinity for *Pf*TyrRS with a K_D_ value of (2.9 ± 1.4) × 10^−7^ M (Figure 5F). Additionally, we employed a limited proteolysis analysis to investigate protein structure and conformational changes. This method involves treating the protein with trypsin, a specific protease enzyme, to cleave it predominantly at Arg or Lys sites, generating smaller fragments. Our results indicate that *Pf*TyrRS bound to okanin showed a reduced susceptibility to proteolysis compared to unbound *Pf*TyrRS, suggesting potential conformational changes induced by okanin (Figure 6A). However, we observed no significant difference or even greater susceptibility to limited proteolysis of *Hs*TyrRS between the control and okanin-added groups (Figure 6B).

### 2.5. Potential Mechanism of PfTyrRS Inhibition by Okanin

**Figure 7:**
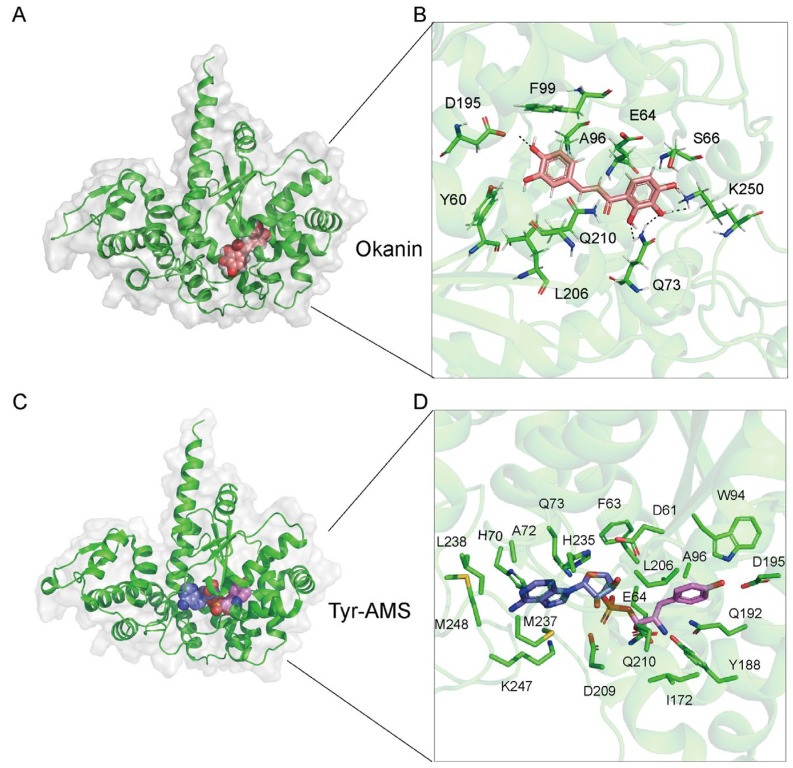
The structure of *Pf*TysRS combined with okanin and Tyr-AMS, respectively. (**A**) The molecular docking model of *Pf*TysRS and okanin was generated by Autodock Vina v.1.2.2 and PyMol (https://pymol.org, accessed on 20 March 2024). (**B**) Okanin was located in the catalytic pocket of the *Pf*TysRS and formed interaction forces with multiple amino acid residues, such as Y60, E64, S66, Q73, A96, F99, D195, L206, Q210, and K250. The structure of *Pf*TysRS is shown as a green cartoon; okanin is shown as pink stick. (**C**) The structure of *Pf*TysRS and Tyr-AMS (PDB: 3VGJ) was generated by Pymol (https://pymol.org, accessed on 20 March 2024). (**D**) Tyr-AMS formed interaction forces with multiple amino acid residues. The structure of *Pf*TysRS is shown as a green cartoon; Tyr-AMS is shown as blue and purple sticks.

To further investigate the mechanism underlying the inhibition of *Pf*TyrRS by okanin, we performed a molecular docking analysis of okanin and *Pf*TyrRS based on the structure of *Pf*TyrRS (Figure 7A). We aimed for a reliable docking model, with a lower binding energy indicating a more stable binding between the ligand and the receptor. The docking structure score was −7 kcal/mol, indicating a reliable model. Docking on the full *Pf*TyrRS protein with okanin resulted in a score of −8.595 kcal/mol, with a preference observed for the catalytic region as the binding site. The main forces involved are hydrophobic force, hydrogen bonds, and aromatic ring stacking (Figure 7A). Similarly, the docking on the *Hs*TyrRS protein produced a score of −7.738 kcal/mol. The docking outcomes revealed that the ligand was situated in the catalytic region. We also investigated the potential binding mode between *Pf*TyrRS and okanin. The ligand okanin interacts with 10 residues of *Pf*TyrRS, Y60, E64, S66, Q73, A96, F99, D195, L206, Q210, and K250 (Figure 7B). The interaction forces between Y60, S66, Q73, D195, Q210, and K250 of *Pf*TyrRS with okanin are hydrogen bonds. Additionally, a hydrophobic force is formed between E64, A96, F99, and L206 of *Pf*TyrRS and okanin. Furthermore, F99 of *Pf*TyrRS forms an aromatic ring stacking interaction with okanin.

Tyr-AMS, which mimics the intermediate products of ATP and tyrosine, binds to the same pocket as a substrate for *Pf*TyrRS (Figure 7C). Within this pocket, 20 amino acids in *Pf*TyrRS interact with Tyr-AMS (Figure 7D). Notably, five amino acids, E64, Q73, A96, L206, and Q210, can interact with both Tyr-AMS and okanin. As a result, okanin occupies the substrate Tyr-AMS site, effectively preventing the substrate from binding to the enzyme.

### 2.6. Okanin Binds to the Tyrosine Binding Site and ATP Binding Region of PfTyrRS

**Figure 8:**
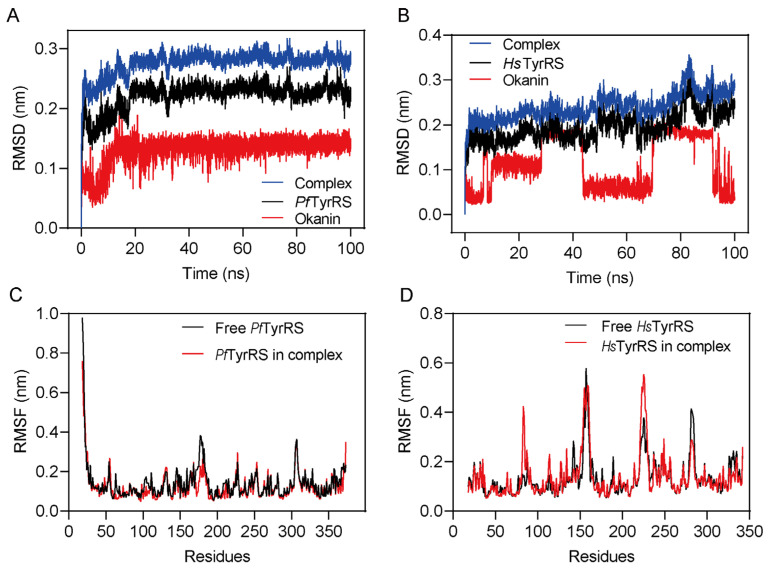
Molecular dynamics simulation. (**A**) The MD simulation (RMSD analysis) of *Pf*TysRS/okanin complexes for 100 ns. (**B**) The MD simulation (RMSD analysis) of *Hs*TysRS/okanin complexes for 100 ns. (**C**) The MD simulation (RMSF analysis) of *Pf*TysRS/okanin complexes for 100 ns. (**D**) The MD simulation (RMSF analysis) of *Hs*TysRS/okanin complexes for 100 ns.

Molecular dynamics (MD) simulations can assist in analyzing the structural dynamics, conformational fluctuations, and stability of protein–ligand complexes. To verify the stability of the complexes formed by okanin with *Pf*TyrRS, we subjected them to an MD simulation analysis. The simulation lasted for 100 ns to analyze the stability and rigidity of the protein–ligand complexes. The stability of the entire complex was analyzed based on the root-mean-square deviation (RMSD) (Figure 8A,B). A root-mean-square fluctuation (RMSF) analysis was used to analyze the conformational fluctuation of the complex (Figure 8C,D). The RMSD value reflects the deviation from the initial conformation to the final conformations of proteins, ligands, and complexes. The results indicate that the ligands maintain good conformational stability in the *Pf*TyrRS/okanin complexes throughout the simulation process (Figure 8A). However, the conformational stability of the ligands in the *Hs*TyrRS/okanin complex was poor (Figure 8B). The RMSF analysis identifies the flexible regions of the protein–ligand complexes. The RMSF value determines the movement of each residue within the protein–ligand complex. A higher RMSF value indicates that the region of the complex is more flexible, such as loops, beta-turns, and random coils. We calculated the RMSF to predict the degree of protein structural changes caused by binding ligands. The results show that the RMSF value of the *Pf*TyrRS/okanin complex is below 0.3 nm, indicating that the binding of okanin to *Pf*TyrRS is stable (Figure 8C). The higher RMSF value observed in the *Hs*TyrRS/okanin complex compared to the *Pf*TyrRS/okanin complex suggests that the binding stability between okanin and *Hs*TyrRS is low (Figure 8D).

**Figure 9:**
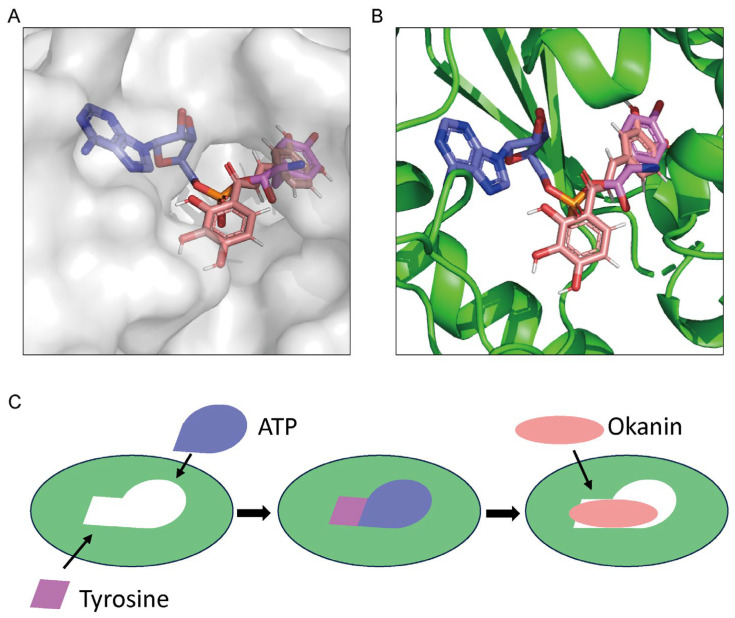
Proposed mechanism for *Pf*TysRS competition. (**A**) The structure of *Pf*TysRS in complex with okanin and Tyr-AMS. The protein is shown as a surface in gray, okanin is presented as pink sticks, ATP is presented as blue sticks, and tyrosine is presented as purple sticks. (**B**) The *Pf*TysRS is shown as a cartoon in green, and okanin is presented as pink sticks, ATP is presented as blue sticks, and tyrosine is presented as purple sticks. (**C**) Schematic of competition mechanism.

To gain a better understanding of the competitive binding mechanism of okanin, we created a Schematic drawing. The surface density map (Figure 9A) and cartoon map (Figure 9B) confirmed that okanin binds to the tyrosine binding site and a portion of the ATP binding site of *Pf*TyrRS. This binding prevents tyrosine and ATP from entering the *Pf*TyrRS binding pocket, thereby inhibiting the activity of *Pf*TyrRS in ATP hydrolysis (Figure 9C).

### 2.7. Okanin Inhibited Plasmodium Growth

**Figure 10:**
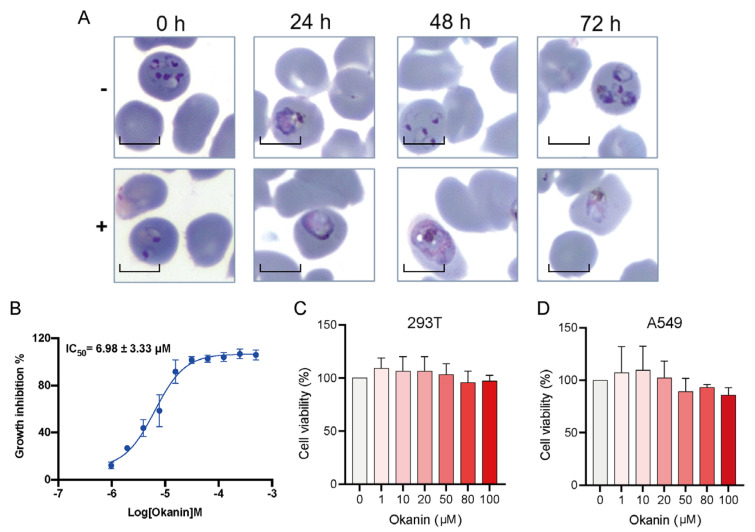
Okanin suppressed the growth of erythrocytic-stage parasites. (**A**) The specific time of action for okanin in erythrocytic-stage parasites (strain 3D7) was determined by treating synchronized parasites and monitoring the cultures over a 72 h period. The morphology of untreated parasites (–) and parasites treated with 11 μM okanin (+) were monitored by Giemsa-stained thin blood smears. Scale bars: 5 μm. (**B**) Okanin inhibits the growth of erythrocytic-stage parasites. Error bars represent the SD of three biological repeats. (**C**) The cell viability of 293T cells treated with different concentrations of okanin was measured by CCK-8 for 24 h. (**D**) The cell viability of A549 cells treated with different concentrations of okanin was measured by CCK-8 for 24 h.

To determine the inhibitory effect of okanin on *Plasmodium* growth, *P. falciparum* 3D7 parasites were synchronized to the ring stage and treated with okanin, and parasite maturation was monitored over 72 h. The parasites treated with solvent control could reproduce asexually, and the transition from trophozoite to schizont was observed in the Giemsa-stained blood smears. However, parasites treated with okanin stopped growing completely until they eventually died (Figure 10A). We further evaluated the inhibitory potency of okanin on *P. falciparum* strain 3D7 in the red stage. In this experiment, parasite growth is determined by using SYBR Green I, a dye with marked fluorescence enhancement upon contact with *Plasmodium* DNA. The IC_50_ was 6.98 ± 3.33 μM (Figure 10B), confirming the antimalarial activity of okanin. Furthermore, we assessed okanin’s cytotoxicity using CCK-8. Treatment with different concentrations of okanin for 24 h resulted in 293T and A549 cell viabilities above 85% even at 100 µM of okanin (Figure 10C,D). These findings collectively demonstrate okanin’s efficacy in inhibiting *Pf*TyrRS activity and parasite growth, with low toxicity.

## 3. Discussion

AaRSs are indispensable enzymes involved in translation, essential for cellular life across various organisms. Inhibitors of aaRSs are commonly used as antimicrobial agents or to combat parasitic infections [20,21,22]. *Pf*TyrRS, a target for antimalarial drug development, emerges as promising due to a comparative analysis of amino acid sequences and three-dimensional structures of human and *Plasmodium* TyrRS. Recent studies have shown that ML901 is effective and selective in murine models of malignant malaria [15]. This underscores the importance of *Pf*TyrRS as a viable drug target. The aim of this study was to target *Pf*TyrRS to identify potential new drugs against *P. falciparum* infection.

In this study, we successfully expressed and purified *Pf*TyrRS and *Hs*TyrRS proteins, confirming their ATP hydrolysis activities through enzyme activity experiments. Subsequently, we screened 2141 compounds using the TSA assay, identifying nine compounds that altered the Tm value of *Pf*TyrRS. Notably, only okanin exhibited significant inhibitory activity against ATP hydrolysis, suggesting that the other compounds may have low affinity or ineffective binding to the catalytic pocket. Furthermore, BLI confirmed the high affinity of okanin for *Pf*TyrRS, while MD results demonstrated stable interactions between okanin and *Pf*TyrRS. Molecular docking revealed that okanin occupied the Tyr-AMS binding site, engaging with *Pf*TyrRS through hydrogen bonding, hydrophobic forces, and π-π stacking. These findings highlight okanin as a promising candidate for the development of antimalarial drugs.

Although our study yielded significant findings, there are limitations that need to be addressed. Firstly, while okanin demonstrated a notable effect on suppressing ATP hydrolysis activity in in vitro experiments, further investigations are necessary to evaluate its preventive and therapeutic effects against *P. falciparum* in cellular or animal models. Additionally, okanin has been reported to significantly suppress LPS-induced iNOS expression and inhibit IL-6 and TNF-α production and mRNA expression in LPS-stimulated BV-2 cells [23]. The potential additional biological functions of okanin in the human body remain unknown. It is important to note that okanin and its derivatives have not yet been approved for clinical use. Therefore, comprehensive analyses of their safety profile, oral bioavailability, and other essential drug evaluation parameters are imperative before considering them for clinical applications.

In summary, malaria is one of the greatest human killers in history and remains a major public health problem. Studies have shown widespread parasite resistance to currently used therapies [24], as well as mosquito vector resistance to pyrethroid insecticides [25]. In particular, the recent emergence of artemisinin-resistance-conferring K13 mutations in Africa is of great concern [26,27]. There is an urgent need to develop new antimalarial compounds with novel mechanisms of action. Since the protein structure of *Pf*TyrRS is different from that of *Hs*TyrRS [15], we screened natural bioactive products that can significantly suppress the ATP hydrolyzing activity of *Pf*TyrRS. Thus, our results showed that okanin or its derivatives may be promising candidates for the treatment of diseases caused by *P. falciparum* infection.

## 4. Materials and Methods

### 4.1. Comparative Sequence Analysis of TyrRS

The homologous TyrRS sequences of 4 species, i.e., *Plasmodium falciparum* (*P. falciparum*), *Homo sapiens* (*H. sapiens*), *Bos taurus* (*B. taurus*), and *Pongo abelii* (*P. abelii*), were fetched from the protein reference database of NCBI. The sequences were analyzed using Bioedit v.7.0.9 software. Alignments were generated using ESPript (https://espript.ibcp.fr/, accessed on 20 March 2024).

### 4.2. Preparation of Recombinant Proteins PfTyrRS and HsTyrRS

The *Pf*TyrRS gene (ID: PF3D7-0807900) was synthesized and cloned into the pET28a vector, including a C-terminal 10 × His tag. Constructs were transformed into BL21 cells. Cultures were grown overnight to saturation in a lysogeny broth (LB) medium containing 50 µg/mL of ampicillin. The overnight culture was diluted 1/100 in LB medium and grown at 37 °C. Isopropyl ß-d-thiogalactopyranoside was added to a final concentration of 0.5 mM at 600 OD of 0.9, and then the cells were grown for 20 h at 16 °C. The cells were collected by centrifugation at 8000 rpm for 40 min. The pellet was resuspended in lysis buffer (20 mM Tris-HCl pH 7.5, 300 mM NaCl, 5 mM imidazole, and 1 mM phenyl-methyl-sulphonyl-fluoride). The cells were disrupted by an ultrasonic cell crusher and the lysate was clarified by centrifugation at 20,000 rpm for 45 min. The proteins were purified using Ni-NTA beads (Cytiva, Shanghai, China), a HiTrap Q HP column (Cytiva, Shanghai, China), and a HiLoad 16/60 Superdex 200 Increase 10/300 GL grade column (Cytiva, Shanghai, China). All purification steps were carried out at 4 °C or on ice. The correctly identified *Pf*TyrRS and *Hs*TyrRS proteins were stored at −80 °C. *Hs*TyrRS was constructed in the vector pET28a with a C-terminal 10 × His tag and purified similarly. The protein concentrations used in all the experiments were determined by the Bradford method [28].

### 4.3. Thermal Shift Assay

The dye from the Protein Thermal Shift™ Kit (Thermo Fisher Scientific, Waltham, MA, USA) was used to monitor the thermal stability of the protein by binding to the exposed hydrophobic regions. Thermal shift assays were performed on a StepOnePlus 7 Flex Real-Time Cycler (Applied Biosystems, Pleasanton, CA, USA). For compound screening, solutions of 14 μL of protein thermal shift buffer (40 mM Hepes, pH 7.5, 200 mM NaCl), 2 µL of diluted thermal shift dye (125×), 2 µL of protein at 2 mg/mL, and 2 µL of natural bioactive products (TargetMol, Boston, MA, USA) were added to the wells of 96-well optical reaction plates (Applied Biosystems, Pleasanton, CA, USA). The 96-well polymerase chain reaction plates were placed in the system (Life Technologies, Carlsbad, CA, USA), incubated at room temperature for 30 min, and gradually heated from 25 °C to 95 °C at a rate of 1 °C/min. The fluorescence signal of SYPRO orange at 490/530 nm excitation/emission wavelengths during protein thermal denaturation was recorded by the instrument every 30 s. The melting temperature (Tm) of the protein was measured according to the melting curves of the protein (fluorescence intensity vs. temperature). DMSO was used as a negative control. The ΔTm is the shift in the value between the melting temperatures of *Pf*TyrRS with the compound and the negative control. The fluorescence signal was monitored and plotted versus the temperature, and the midpoint of the protein unfolding transition is defined as the Tm.

### 4.4. ATP Hydrolysis Assay

The ATP consumption levels were determined using a luciferin/luciferase assay according to the protocol of the ATP assay kit (Beyotime, Shanghai, China). The luminescence reaction temperature was set internally to 37 °C for 50 min, once every minute. The total reaction system was 20 µL. First, the ATP detection reagent was diluted 25 times with diluent buffer (50 mM Tris-HCl pH 7.6, 50 mM KCl, 25 mM MgCl_2_, 0.1 mg/mL BSA, 1 mM DTT), and 10 µL was added to a 384 white board. Then, ATP, amino acids, and TyrRS were mixed in equal volume, and then 10 µL was quickly mixed and added to the 384 white board to react with the ATP detection reagent. The luminescence value was measured in real time by an enzyme marker. The final concentration of L-tyrosine was 200 µM and the final concentration of ATP was 10 µM. Enzyme activity inhibition experiments were performed to add small-molecule drugs to the above reaction conditions. First, 1.25 µM *Pf*TyrRS or 1 µM *Hs*TyrRS was incubated with different small-molecule-drug concentrations at room temperature for 30 min. Then, the ATP detection reagent was added to the 384 white board, and finally the mixture of TyrRS and the small-molecule drug was quickly mixed with ATP and L-tyrosine and added to the ATP detection reagent. The luminescence reaction temperature was set internally to 37 °C for 50 min.

### 4.5. BLI Analysis for Interactions of Okanin and PfTyrRS

An Octet Red 96 system (ForteBio, Gottingen, Germany) was used to detect interactions between okanin and *Pf*TyrRS using Ni-NTA biosensors according to the standard instructions with minor modifications. Black 96-well plates (ForteBio, Gottingen, Germany) were used for all assays. Ni-NTA sensors were pre-wetted with PBS for at least 10 min before each assay. The running volume for all samples or buffers was 200 µL. The rpm option and plate temperature were kept at 1000 rpm and 37 °C, respectively, for the entire assay. Affinity measurements were carried out for *Pf*TyrRS by *Pf*TyrRS on Ni-NTA sensors and subjecting them to binding with okanin. Standard solutions of okanin at different concentrations (20, 10, 5, 2.5, and 1.25 μM) were used. First, the Ni-NTA biosensors were equilibrated with PBS for 120 s, followed by immobilization of the *Pf*TyrRS (4 µM in PBS, 300 s). Then, the different concentrations of okanin were recorded for 250 s. The dissociation step was performed in PBS for 150 s.

### 4.6. Molecular Dynamics Simulation

A protein molecule of *Pf*TyrRS and a small molecule of okanin make up the system used in this experiment, which is called a protein–small molecule complex. For the simulation, we used the Gromacs program, and the steps involved are as follows. First was the molecular preparation. Using the pdb2gmx and gmx editconf commands, we created topological files and simulation boxes after importing the structure files of small molecules and proteins into Gromacs, respectively. Next was the energy minimization. Using the gmx grompp and gmx mdrun commands, we reduced the structural bulk of proteins and small molecules. Then, the MD simulation was performed. We used the gmx grompp and gmx mdrun commands to model the MD of proteins and small molecules for up to 100 ns and recorded the conformational information during the simulation. The simulation conditions were conducted at a static temperature of 300 K and atmospheric pressure (1 bar). The Amber99sb-ildn force field was utilized, with water molecules serving as the solvent (Tip3p water model). The volume of the *Pf*TyrRS box was 397.69 nm^3^ (x: 6.249 nm, y: 5.295 nm, and z: 7.020 nm). And the volume of the *Hs*TyrRS box was 330.39 nm^3^ (x: 5.916 nm, y: 4.901 nm, and z: 6.410 nm). Finally, the data analysis was performed. We used the gmx rms command to calculate the RMSD, the RMSF, and other indicators between proteins and small molecules, and presented and analyzed the results by drawing pictures and statistical tables.

### 4.7. Molecular Docking

Autodock Vina v.1.2.2 software was used for molecular docking [29]. The structure of the *Pf*TyrRS receptor macromolecule and ligand okanin were prepared before docking. The macromolecule receptor was modified by removing water molecules and adding polar hydrogen atoms, and then the semiflexible docking method was used to generate the model. Molecular visualization and analysis were performed using PyMol (https://pymol.org, accessed on 20 March 2024).

### 4.8. In Vitro Plasmodium Growth Assay

Parasites cultured in human O^+^ erythrocytes followed standard procedures. To obtain >80% ring-stage parasites, asynchronous cultures were pretreated with 5% sorbitol. *P. falciparum* strain 3D7 at the mid-ring stage (6–10 h post-invasion) was used for antimalarial testing in 96-well plates. Each well contained 1% parasitemia and 2% hematocrit in a total volume of 200 µL. Compounds were serially diluted from 512 µM with 2-fold gradient dilution, resulting in 10 concentrations. DMSO served as the negative control (NC), and cultured erythrocytes without *Plasmodium* served as the positive control (PC). After 72 h of incubation at 37 °C with 5% CO_2_, 5% O_2_, and 90% N_2_, fluorescence was measured at 485 nm excitation and 535 nm emission. The % inhibition was calculated as (NC-fluorescence) × 100/(NC-PC). For the morphological evaluation, ring-stage parasites were treated with 11 µM of the compound in a total volume of 10 mL, with DMSO as the control. Giemsa-stained blood smears were examined at 0, 24, 48, and 72 h post-treatment using a microscope.

### 4.9. Cytotoxicity Test

Cell viability was analyzed by Cell Counting Kit-8 (CCK-8, Biosharp, Beijing, China) according to the manufacturer’s protocols. Cells were seeded and cultured at a density of 5 × 10^3^/well in 100 µL of medium into 96-well microplates (Labselest, Beijing, China). Then, the cells were treated with various concentrations of okanin (0, 1, 10, 20, 50, 80, and 100 μM). After treatment for 24 h, 10 µL of the CCK-8 reagent was added to each well and then cultured for 1 h. The absorbance was analyzed at 450 nm using a microplate reader (Bio-Rad, Hercules, CA, USA) using wells without cells as blanks. The proliferation of cells was expressed by the absorbance. All experiments were performed in triplicate.

## Figures and Tables

**Table 1:** The nine positive hits of the thermal shift assay and their inhibitory effect on *Pf*TyrRS.

Compound	Structure	ΔTm [°C]	Relative Enzyme Activity [%]
Okanin	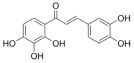	−4.0	31.1
Baccatin III	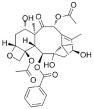	+2.8	91.9
Cardamonin	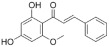	−3.1	88.5
Citric acid	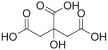	+3.9	95.4
Quercetin	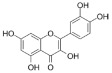	+2.6	72.7
Quercetagetin	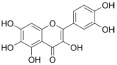	−2.9	69.3
Dryocrassin ABBA	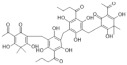	−4.1	76.5
6-Ethoxydihydrosanguinarine	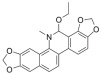	−3.8	75.9
Iberverin	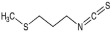	−2.8	67.5

## Data Availability

Data is contained within the article and Supplementary Materials.

## Supplementary Materials

The following supporting information can be downloaded at: https://www.mdpi.com/article/10.3390/ijms25094751/s1.
